# Silicon quantum dot superlattice solar cell structure including silicon nanocrystals in a photogeneration layer

**DOI:** 10.1186/1556-276X-9-246

**Published:** 2014-05-20

**Authors:** Shigeru Yamada, Yasuyoshi Kurokawa, Shinsuke Miyajima, Makoto Konagai

**Affiliations:** 1 Department of Physical Electronics, Tokyo Institute of Technology, 152-8552 Meguro-ku, Tokyo, Japan; 2 Photovoltaics Research Center (PVREC), Tokyo Institute of Technology, 152-8552 Meguro-ku, Tokyo, Japan

**Keywords:** Silicon nanocrystals, Silicon quantum dot, Solar cells, Quantum size effect

## Abstract

The solar cell structure of n-type poly-silicon/5-nm-diameter silicon nanocrystals embedded in an amorphous silicon oxycarbide matrix (30 layers)/p-type hydrogenated amorphous silicon/Al electrode was fabricated on a quartz substrate. An open-circuit voltage and a fill factor of 518 mV and 0.51 in the solar cell were obtained, respectively. The absorption edge of the solar cell was 1.49 eV, which corresponds to the optical bandgap of the silicon nanocrystal materials, suggesting that it is possible to fabricate the solar cells with silicon nanocrystal materials, whose bandgaps are wider than that of crystalline silicon.

**PACS:**

85.35.Be; 84.60.Jt; 78.67.Bf

## Background

Over the past few years, many researchers have shown an interest in silicon nanostructures, such as silicon nanocrystals [1-4] and silicon nanowires [5-8] for solar cell applications. Since a silicon nanocrystal embedded in a barrier material can make carriers confined three-dimensionally, the absorption edge can be tuned in a wide range of photon energies due to the quantum size effect. Thus, it is possible to apply silicon nanocrystal materials or silicon quantum dot (Si-QD) materials to all silicon tandem solar cells [9], which have the possibility to overcome the Shockley-Queisser limit [10]. Moreover, it has been found that the weak absorption in bulk Si is significantly enhanced in Si nanocrystals, especially in the small dot size, due to the quantum confinement-induced mixing of Γ-character into the *X*-like conduction band states [11]. Therefore, Si-QD materials are one of the promising materials for the third-generation solar cells. Size-controlled Si-QDs have been prepared in an amorphous silicon oxide (a-SiO_2_) [12], nitride (a-Si_3_N_4_) [13], carbide (a-SiC) [14-17], or hybrid matrix [18,19], which is called as silicon quantum dot superlattice structure (Si-QDSL). In the case of solar cells, generated carriers have to be transported to each doping layer. Since the barrier height of an a-SiC matrix is relatively lower than that of an a-Si_3_N_4_ or a-SiO_2_ matrix, the Si-QDSL using an a-SiC matrix has an advantage in carrier transport. Therefore, the development of the Si-QDSL solar cells using an a-SiC matrix is of considerable importance. There are a few researches fabricating Si-QDSL solar cells. Perez-Wurfl et al. reported that Si-QDSL solar cells with SiO_2_ matrix showed an open-circuit voltage (*V*_oc_) of 492 mV. However, the clear evidence of the quantum size effect has not been reported from Si-QDSL solar cells [20]. In our previous work, Si-QDSLs with a-SiC matrix have been prepared by plasma-enhanced chemical vapor deposition (PECVD). The defect density in a Si-QDSL has been successfully reduced by hydrogen plasma treatment (HPT), and the shift of photoluminescence spectra from the Si-QDSLs has been confirmed by varying the diameter of the Si-QDs [2]. Moreover, it has been revealed that the oxygen-incorporation into the a-SiC matrix can suppress the formation of the leakage paths [21]. An *V*_oc_ of 518 mV has been obtained in a Si-QDSL solar cell with an amorphous silicon oxycarbide (a-Si_1 - *x* - *y*_C_*x*_O_*y*_) matrix [1].

In this paper, we report the effect of oxygen addition on the formation of Si-QDs in a-Si_1 - *x* - *y*_C_*x*_O_*y*_. Optical absorption coefficients of the Si-QDSL were also investigated. Si-QDSL solar cells were fabricated using the optimum oxygen concentration. In addition, the numerical analysis using the Bohm quantum potential (BQP) method was performed to simulate the electrical characteristics of fabricated solar cells.

## Methods

### Experimental method

The a-Si_1 - *x* - *y*_C_*x*_O_*y*_ matrix was deposited on a quartz substrate to investigate the fundamental optical properties such as Raman scattering spectrum, transmittance, and reflectance. The fabrication method is referred as follows. A 40-period-multilayer with silicon-rich hydrogenated amorphous silicon oxycarbide layers and hydrogenated amorphous silicon oxycarbide barrier layers was prepared on a quartz substrate by very high frequency PECVD method (VHF-PECVD). The source gases were silane (SiH_4_), monomethylsilane (MMS), hydrogen (H_2_), and carbon dioxide (CO_2_). The flow rates of SiH_4_, MMS, and H_2_ + CO_2_; deposition pressure; substrate temperature; frequency; and plasma power were fixed at 3.3 , 1.3, and 47.4 sccm; 20 Pa; 60 MHz; 193 °C; and 13 mW/cm^2^, respectively. The flow rate of CO_2_ was varied from 0 to 3.7 sccm. The mass flow controllers for SiH_4_ and CO_2_ were calibrated by N_2_. A H_2_-calibrated mass flow controller was used for MMS. During the deposition of a-Si_1 - *x* - *y*_C_*x*_O_*y*_ barrier layers, the flow of SiH_4_ gas was stopped. Subsequently, the samples were annealed at 900 °C for 30 min under a forming gas atmosphere to form Si-QDs in an a-Si_1 - *x* - *y*_C_*x*_O_*y*_ matrix. The target size of Si-QDs and barrier width were 5 and 2 nm, respectively. The concentrations of Si, C, and O in the barrier layer were measured by X-ray photoelectron spectroscopy (XPS). The crystallinity of Si-QDs was investigated by Raman scattering spectroscopy. The absorption coefficient of a Si-QDSL was estimated by the transmittance and the reflectance of a sample. The samples with uniform thickness were selected for the measurements, and one measurement was carried out for each measurement method and for each sample.

**Figure 1:**
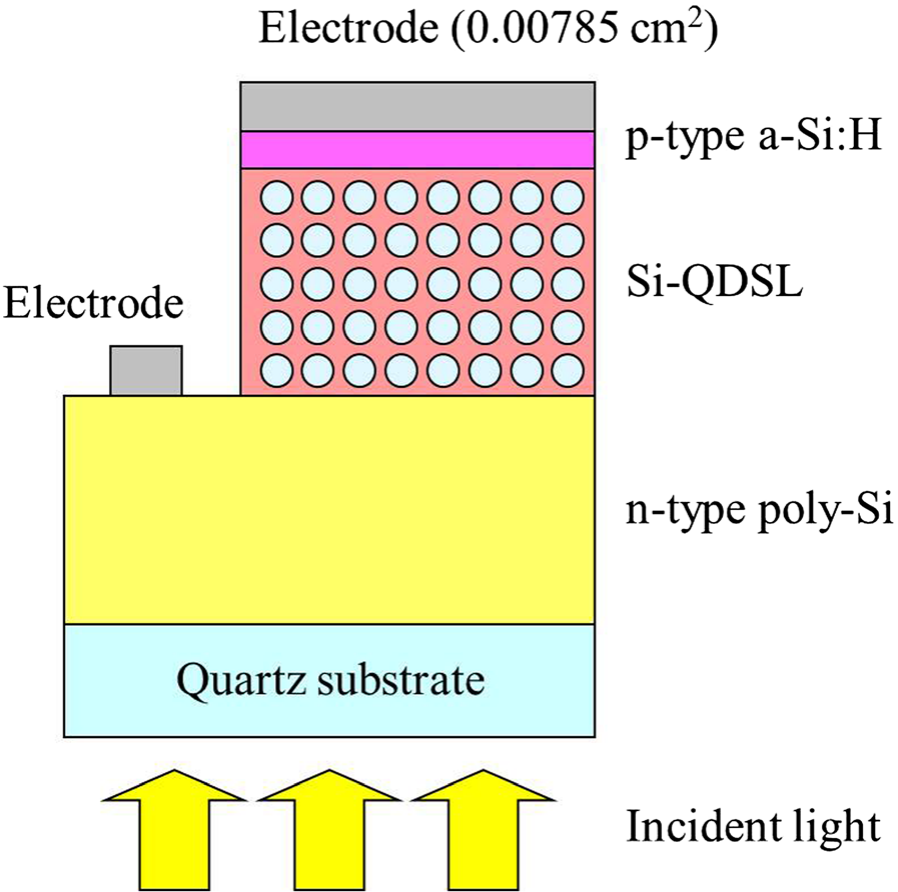
The schematic of the fabricated Si-QDSL solar cell structure.

The solar cells using Si-QDSL as an absorber layer were also fabricated. The schematic of the solar cell structure is shown in Figure 1. The fabrication process is referred as follows. A phosphorus-doped hydrogenated amorphous silicon thin film was deposited on a quartz substrate by PECVD. The film was annealed at 900°C for 30 min under a forming gas, resulting in a polycrystalline silicon (n-type poly-Si) thin film. On the poly-Si layer, a 30-period superlattice was deposited by VHF-PECVD. The same deposition conditions as referred to above were used except the flow rates of CO_2_ and H_2_, which were fixed at 0.4 and 47.0 sccm, respectively. Consequently, the sample was annealed at 900°C for 30 min to form Si-QDs. The sample was exposed to hydrogen plasma to reduce dangling bond defects in the post-annealed Si-QDSL. After the treatment, a boron-doped hydrogenated amorphous silicon (p-type a-Si:H) was deposited by PECVD. An aluminum (Al) electrode was deposited by the evaporator on the sample. The electrode area of a solar cell was 0.00785 cm^2^. The cross-sectional structure of a solar cell was observed by transmission electron microscopy (TEM). The solar cells were characterized by dark *I*-*V* characteristics and light *I*-*V* characteristics under the illumination at AM1.5G, 100 mW/cm^2^, and 25°C.

### Numerical method

**Figure 2:**
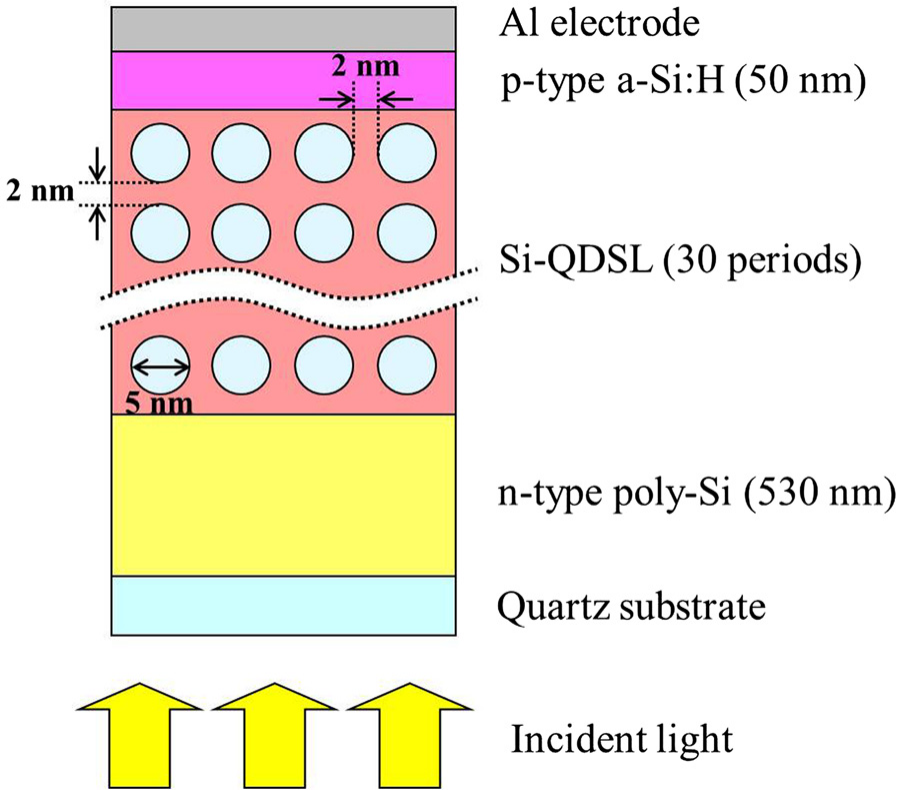
The structure of the Si-QDSL solar cell for numerical calculations.

The numerical calculations of the Si-QDSL solar cells were performed using a two-dimensional device simulator, Atlas ver. 5.18.3.R (Silvaco, Inc., Santa Clara, CA, USA). The device structure used for numerical calculations is shown in Figure 2. Quartz substrate/n-type poly-Si/30-period Si-QDSL (Si-QDs embedded in a-Si_1 - *x* - *y*_C_*x*_O_*y*_)/p-type hydrogenated amorphous silicon (p-type a-Si:H)/Al electrode structure was assumed in this simulation. The diameter of Si-QDs and the gap between any two Si-QDs were fixed at 5 and 2 nm, respectively. The BQP method [22-26] was adopted to describe the quantum confinement effect and the quantum tunnel effect in the Si-QDSL layer. The electrical transport in the Si-QDSL was described by drift-diffusion equations and current continuity equations for electrons and holes. In the theory, the transport of carriers is influenced by the total potential of the potential characterizing the system and quantum potential. The definition of the effective quantum potential *Q*_eff_ is derived from a weighted average of the BQPs seen by all single-particle wavefunctions, which can be expressed as

(1) $$Q_{\mathrm{eff},\;n}=-\gamma_{n}\frac{h^{2}}{8\pi^{2}m_{n}}\frac{\nabla^{2}\sqrt{n}}{\sqrt{n}}\;$$

and

(2) $$Q_{\mathrm{eff},\;p}=-\gamma_{p}\frac{h^{2}}{8\pi^{2}m_{p}}\frac{\nabla^{2}\sqrt{p}}{\sqrt{p}}$$

where *Q*_eff,*n*_ and *Q*_eff,*p*_ are the effective BQPs for the conduction band and the valence band, respectively. *h* is Planck's constant. *n* and *p* are the electron and hole concentrations, respectively. *γ*_*n*_ and *γ*_*p*_ are adjustable parameters for quantum confinement. In general, a three-dimensional quantum system cannot be described on a two-dimensional device simulator due to the difference of the quantum confinement effects between two- and three-dimensional systems. To take three-dimensional quantum effect into two-dimensional simulation, we adjusted the *γ*_*n*_ and *γ*_*p*_ parameters in the BQP model. The parameter values were determined to satisfy that the bandgap calculated from the BQP method is equal to the bandgap derived from three-dimensional Schrödinger equations. The *γ*_*n*_ and the *γ*_*p*_ for the Si-QDSL with 5-nm-diameter Si-QDs and 2-nm-thick a-Si_1 - *x* - *y*_C_*x*_O_*y*_ barrier layers were 4.0. In this simulation, the radiative recombinations, Shockley-Read-Hall (SRH) recombination [27-29] and Auger recombination, were taken into account. Auger coefficients and effective masses of bulk Si were adapted for all layers. The other parameters are shown in Table 1. The bandgaps in the table do not affect optical absorption but carrier transport phenomenon. To take into account the phosphorus diffusion into the Si-QDSL layer, a calculation with the donor concentration in Si-QDs of 1 × 10^17^ cm^-3^ was also performed. The light *I*-*V* characteristics were calculated, assuming solar illumination of AM1.5G at 100 mW/cm^2^. Additionally, the quantum efficiencies were calculated without bias light and bias voltage. An incident light was put into the solar cells from the quartz substrate side normally. The light intensity and the photogeneration rate were calculated based on the ray tracing method, where the Si-QDSL was regarded as an optically homogeneous material, and the optical parameters from the spectroscopic ellipsometry measurement of the Si-QDSL were used.

**Table 1:** Parameters of each layer for calculations

**Parameters**	**n-type poly-Si**	**Si-QD**	**a-Si**_**1 - *x *- *y***_**C**_***x***_**O**_***y***_	**p-type a-Si**
Energy gap (eV)	1.13	1.13	2.5	1.7
Electron affinity (eV)	4.17	4.17	3.5	4.0
Carrier lifetime (s)	1 × 10^-15^	1 × 10^-10^	1 × 10^-10^	1 × 10^-6^
Electron mobility (cm^2^/Vs)	1	1	1	1
Hole mobility (cm^2^/Vs)	0.1	0.1	0.1	0.1
Donor concentration (cm^-3^)	1 × 10^19^	0 or 1 × 10^17^	-	-
Accepter concentration (cm^-3^)	-	-	-	1 × 10^19^

## Results and discussion

### Optical properties of Si-QDSLs

The concentrations of Si, C, and O in a-Si_1 - *x* - *y*_C_*x*_O_*y*_ thin films were measured by the relative sensitivity factor (RSF) method. The concentrations of Si, C, and O for each CO_2_/MMS flow rate ratio were shown in Table 2. The oxygen concentration and the deposition rate of the films depend on the CO_2_/MMS flow rate ratio. The oxygen concentrations of the films prepared without CO_2_ gas and with the CO_2_/MMS flow rate ratios of 0.3, 1.5, and 3.0 were 17.5, 25.1, 32.6, and 39.8 at.%, respectively. Oxygen was observed even in the as-deposited film prepared without flowing CO_2_ gas. This unintentionally incorporated oxygen is thought to be originating from the deposition atmosphere. The deposition rate is proportional to the oxygen concentration in the film, suggesting that the volume of the thin film increases with the oxygen incorporation.

**Table 2:** **Concentrations of Si, C, and O in a-Si**_**1 - *x *- *y***_**C**_***x***_**O**_***y ***_**films with several CO**_**2**_**/MMS flow rate ratios**

**CO**_**2**_**/MMS**	**Si (at.%)**	**C (at.%)**	**O (at.%)**
0	44.6	37.9	17.5
0.3	40.3	34.6	25.1
1.5	34.2	33.2	32.6
3.0	31.9	28.3	39.8

**Figure 3:**
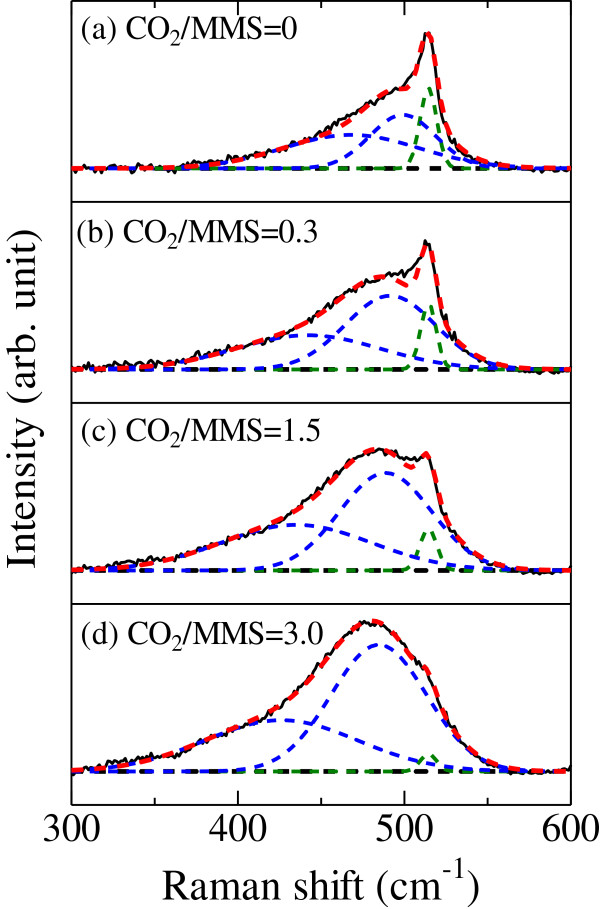
**The Raman spectra of the Si-QDSLs with several CO**_**2**_**/MMS flow rate ratios. (a)** CO_2_MMS = 0. **(b)** CO_2_MMS = 0.3. **(c)** CO_2_MMS = 1.5. **(d)** CO_2_MMS = 3.

The crystallization of Si-QDs was investigated by Raman scattering spectroscopy. The Raman spectra of the Si-QDSLs with the CO_2_/MMS flow rate ratios of 0, 0.3, 1.5, and 3.0 are shown in Figure 3. A Raman spectrum was separated into three Gaussian curves. The peaks at approximately 430 and 490 cm^-1^ are originating from the LO mode and TO mode of a-Si phase, respectively [30]. These Gaussian curves are indicated by blue dashed lines. The peak at approximately 510 cm^-1^ is originating from Si-QDs. The Gaussian curve is indicated by green dashed line. As the CO_2_/MMS flow rate ratio increases, the intensity of the peak from Si-QDs becomes weaker compared with the peak from a-Si phase. This indicates that the crystallization of Si-QDs in the silicon-rich layers is prevented by the oxygen-incorporation, and the crystallization temperature of nanocrystalline silicon phase becomes higher [31].

**Figure 4:**
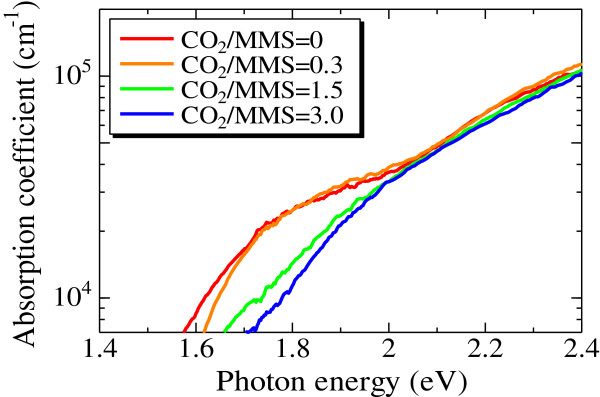
**The absorption coefficients of the Si-QDSLs with several CO**_**2**_**/MMS flow rate ratios.**

The absorption coefficient was estimated from the measurements of transmittance and reflectance. The absorption coefficients of the Si-QDSLs with the CO_2_/MMS flow rate ratios of 0, 0.3, 1.5, and 3.0 are shown in Figure 4. For both Si-QDSLs with the CO_2_/MMS flow rate ratios of 0 and 0.3, the absorption enhancement was observed below the photon energy of 2.0 eV. Moreover, the absorption enhancement becomes weaker as the CO_2_/MMS flow rate ratio increases. This tendency corresponds to that of the intensity of the peak originating from Si-QDs in the Raman scattering spectrum. Therefore, one can conclude that the absorption enhancement is due to the increment of the nanocrystalline silicon phase. Moreover, the absorption edge was estimated by the Tauc model [32]. The absorption edges of the Si-QDSLs with the CO_2_/MMS flow rate ratios of 0 and 0.3 were estimated at 1.48 and 1.56 eV, respectively. These values are similar to the optical gap of 5-nm-diameter Si-QDs in an a-SiC matrix measured by photoluminescence spectrum [2]. On the other hand, the absorption edges of the Si-QDSLs with the CO_2_/MMS flow rate ratios of 1.5 and 3.0 were estimated at approximately 1.70 eV, which corresponds to the optical gap of a-Si.

These results indicate that the CO_2_/MMS flow rate ratio should be below approximately 0.3 to form Si-QDs in the silicon-rich layers. According to the [22], the CO_2_/MMS flow rate ratio should be higher than 0.3 to suppress the crystallization of a-SiC phase in the a-Si_1 - *x* - *y*_C_*x*_O_*y*_ barrier layers and the increment of the dark conductivity for the annealing temperature of 900°C. Although there is a trade-off between the promotion of the crystallization of Si-QDs and the suppression of the crystallization of a-SiC phase, the CO_2_/MMS flow rate ratio of approximately 0.3 or the oxygen concentration of approximately 25 at.% is one of the optimal conditions. Therefore, the CO_2_/MMS flow rate ratio of 0.3 is adopted for the solar cell fabrication in this study.

### *I*-*V* characteristics of the fabricated solar cells

**Figure 5:**
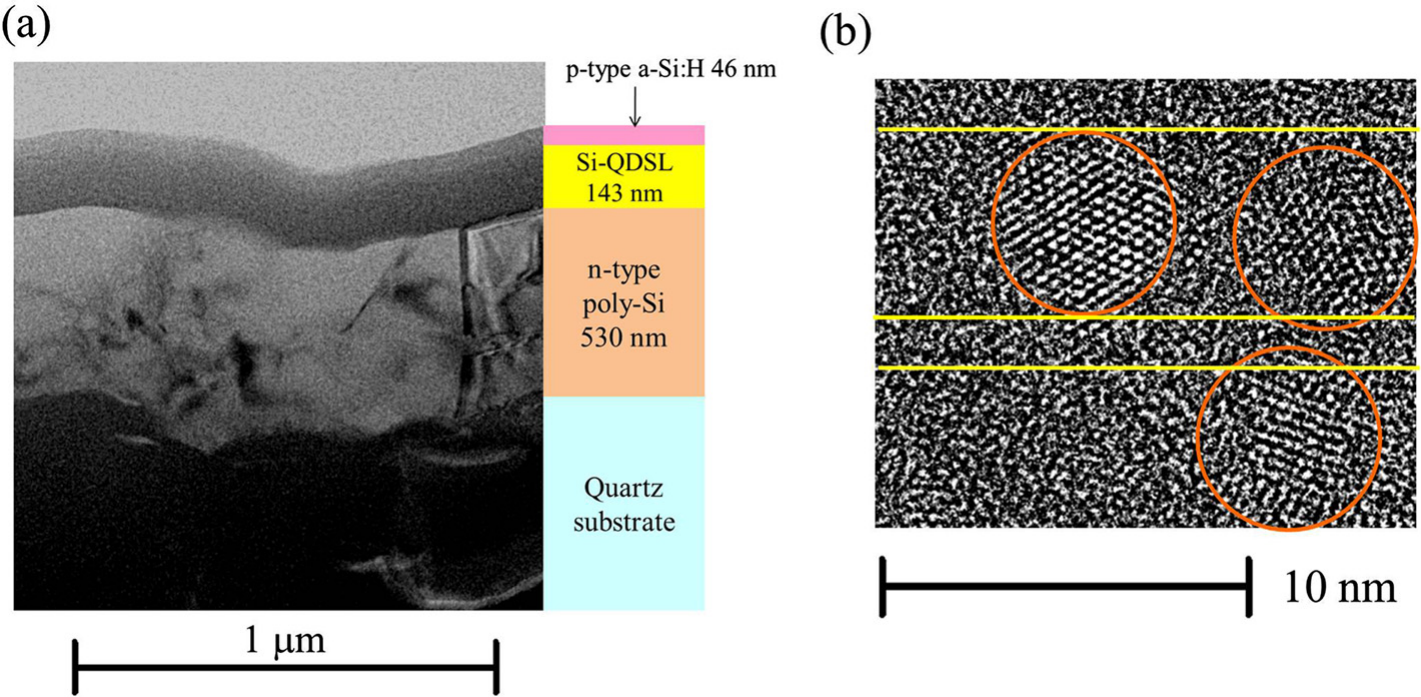
**The cross-sectional TEM images of the fabricated solar cell structure. (a)** The whole region image with the schematic of the structure and the thicknesses of each layer. **(b)** The magnified image of the Si-QDSL layer in the solar cell.

The cross-sectional TEM images of the fabricated solar cell are shown in Figure 5. Figure 5a shows the image of the whole region of the solar cell. Figure 5b shows the magnified image of the Si-QDSL layer in the solar cell. The thicknesses of the n-type poly-Si layer, the Si-QDSL layer, and p-type a-Si:H layer were approximately 530, 143, and 46 nm, respectively. The black region below the n-type poly-Si layer is a quartz substrate. The textured quartz substrate is used to prevent from peeling off the films during the thermal annealing. In Figure 5b, the yellow lines and orange circles indicate the interface between an a-Si_1 - *x* - *y*_C_*x*_O_*y*_ barrier layer and a Si-QD layer, and Si-QDs, respectively. This magnified image revealed that a Si-QDSL layer including average 5-nm-diameter Si-QDs was successfully prepared.

**Figure 6:**
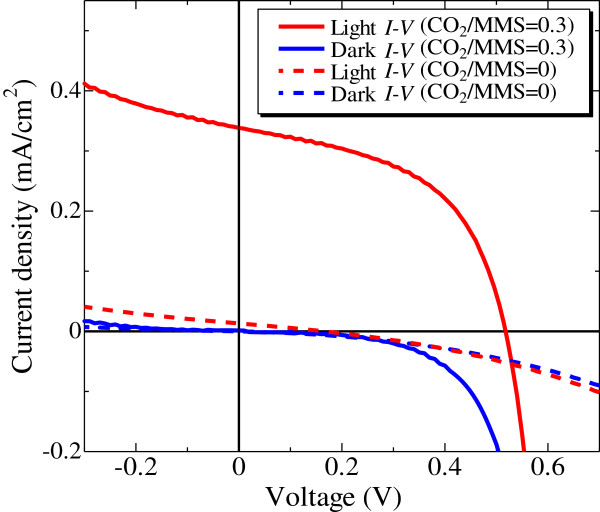
**The *I*-*V *** **characteristics of the fabricated Si-QDSL solar cell**[ [1,3]]**.**

**Figure 7:**
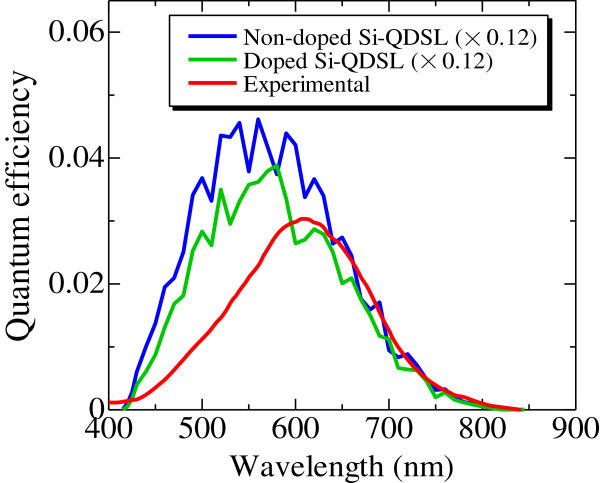
Internal quantum efficiencies of fabricated solar cell and of that calculated by the BQP method.

Figure 6 shows the dark *I*-*V* characteristics and the light *I*-*V* characteristics of the solar cells with the CO_2_/MMS flow rate ratio of 0 and 0.3 [1,3]. The diode properties were confirmed from the dark *I*-*V* characteristics. The characteristics were evaluated by one-diode model:

(3) $$I=I_{0}\exp\left[\frac{q\left(V+R_{s}I\right)}{\mathrm{nkT}}-1\right]-\frac{V+R_{s}I}{R_{\mathrm{sh}}},$$

where *I*_0_, *n*, *R*_s_, and *R*_sh_ represent reverse saturation current density, diode factor, series resistance, and shunt resistance, respectively. According to the fitting of the dark *I*-*V* characteristics of the oxygen-introduced Si-QDSL solar cell, the reverse saturation current density, the diode factor, the series resistance, and the shunt resistance were estimated at 9.9 × 10^-6^ mA/cm^2^, 2.0, 2.3 × 10^-1^ Ω cm^2^, and 2.1 × 10^4^ Ω cm^2^, respectively. The solar cell parameters of the light *I*-*V* characteristics under AM1.5G illumination are summarized in Table 3. An *V*_oc_ of 518 mV was achieved. Compared with the *V*_oc_ of 165 mV with non-oxygen-introduced Si-QDSL solar cells, the characteristics were drastically improved. The possible reasons for this improvement are due to the passivation effect of Si-O phase on silicon quantum dots [33], and the reduction of the leakage current by the introduction of oxygen [21]. Figure 7 shows the internal quantum efficiency of the solar cell. The red line corresponds to the experimental internal quantum efficiency. The quantum efficiency decays to zero at approximately 800 nm, suggesting that the contribution is originating not from the n-type poly-Si but from the Si-QDSL absorber layer.

**Table 3:** Solar cell parameters of the fabricated Si-QDSL solar cells and the calculated by BQP method

**Parameters**	**Experimental**	**Calculated**	
		**Doped Si-QDSL**	**Non-doped Si-QDSL**
*V*_oc_ (mV)	518	520	505
*J*_sc_ (mA/cm^2^)	0.34	3.98	4.96
FF	0.51	0.61	0.69

### Calculations of *I*-*V* characteristics and quantum efficiencies

The light *I*-*V* characteristics and the internal quantum efficiency of the Si-QDSL solar cells with a doped Si-QDSL layer and a non-doped Si-QDSL layer were simulated under AM1.5G illumination using the BQP method. The calculated solar cell parameters are shown in Table 3. Also, the calculated quantum efficiencies are shown in Figure 7. The simulated quantum efficiencies are multiplied by 0.12 for comparison with the experimental one. The calculated short-circuit current densities (*J*_sc_) and quantum efficiencies are much higher than those of the experimental results. There are two possible reasons.

The first reason is due to the difference of the doping concentration in a Si-QDSL layer. In an actual solar cell, the phosphorus concentration in the Si-QDSL absorber layer is more than 1 × 10^19^ cm^-3^ due to the high-temperature annealing process [34]. From the simulations, the *J*_sc_ and the quantum efficiency in the whole wavelength region becomes lower if the phosphorus concentration in the Si-QDSL layer increases. The phosphorus in the Si-QDSL layer degrades the *J*_sc_ due to the reduction of the electrical field in the Si-QDSL layer. Unfortunately, simulations were not possible when the dopant concentration in the Si-QDSL was higher than 1 × 10^17^ cm^-3^ due to the convergence problem of the BQP calculations. It is expected that *J*_sc_ will decrease more if the dopant concentration becomes higher. We previously reported that the quantum efficiency in the whole wavelength region decreases as the dopant concentration in the Si-QDSL increases from experiments and the simulations using classical model [35], which is similar to the results of the BQP method. The second reason is due to the optical losses in the n-type poly-Si layer. In this calculation, the surface roughness of the textured quartz substrate was not taken into account. The effective optical path length in the n-type layer of the simulated structure should be shorter than that of the actual solar cell structure. As a result, the simulated quantum efficiency in the short-wavelength region is higher than that of the experimental because of the low optical absorption loss in the n-type poly-Si layer.

Even though the *J*_sc_ mismatch, the absorption edge can be estimated from the simulated quantum efficiency. The calculated quantum efficiencies at the long-wavelength region are in agreement with those of the experimental one. This suggests that the absorption edge of the solar cell can be theoretically reproduced using this simulation. Moreover, the absorption edge was estimated to be 1.49 eV, which is quite similar to the absorption edge of the Si-QDSL estimated from the optical measurements. This indicates that the photogeneration in the Si-QDSL solar cell is thought to be the contribution from Si-QDs, and it is possible to fabricate the solar cells with silicon nanocrystal materials, whose bandgaps are wider than that of a crystalline silicon.

## Conclusions

The fundamental optical properties of Si-QDSLs were investigated, and the solar cell structure using the Si-QDSL as an absorber layer was fabricated and characterized. From the measurements of the Raman spectra and the absorption coefficients of Si-QDSLs, it was revealed that the absorption coefficient is enhanced by the crystallization of the Si-QDs, and the crystallinity of Si-QDs is affected by the oxygen concentration in the superlattice. In addition, the solar cell characteristics were simulated by the BQP method. The absorption edge of the simulated Si-QDSL solar cell was in agreement with that of the fabricated one. Moreover, the absorption edge of the Si-QDSL solar cell was 1.49 eV, which is similar to the absorption edge estimated from the optical measurements. These results suggest that it is possible to fabricate the solar cells with silicon nanocrystal materials, whose bandgaps are wider than that of a crystalline silicon.

## Competing interests

The authors declare that they have no competing interests.

## Authors' contributions

SY carried out the experiments and the calculations. MK supervised the work and finalized the manuscript. YK and SM participated in the design of the study and the instructions of the calculations, and helped draft the manuscript. All authors read and approved the final manuscript.
